# A fair exchange: why living kidney donors in England should be financially compensated

**DOI:** 10.1007/s11019-023-10171-x

**Published:** 2023-08-24

**Authors:** Daniel Rodger, Bonnie Venter

**Affiliations:** 1 Institute of Health and Social Care, School of Allied and Community Health, London South Bank University, London, UK; 2 Department of Psychological Sciences, University of London, Birkbeck, UK; 3 Centre for Health, Law, and Society, Bristol Law School, University of Bristol, Bristol, UK

**Keywords:** Kidney transplantation, Organ donation, Compensation, Acute kidney failure, Organ grafting, Allogeneic transplantation, Directed organ donation

## Abstract

Every year, hundreds of patients in England die whilst waiting for a kidney transplant, and this is evidence that the current system of altruistic-based donation is not sufficient to address the shortage of kidneys available for transplant. To address this problem, we propose a monopsony system whereby kidney donors can opt-in to receive financial compensation, whilst still preserving the right of individuals to donate without receiving any compensation. A monopsony system describes a market structure where there is only one ‘buyer’—in this case the National Health Service. By doing so, several hundred lives could be saved each year in England, wait times for a kidney transplant could be significantly reduced, and it would lessen the burden on dialysis services. Furthermore, compensation would help alleviate the common disincentives to living kidney donation, such as its potential associated health and psychological costs, and it would also help to increase awareness of living kidney donation. The proposed system would also result in significant cost savings that could then be redirected towards preventing kidney disease and reducing health disparities. While concerns about exploitation, coercion, and the ‘crowding out’ of altruistic donors exist, we believe that careful implementation can mitigate these issues. Therefore, we recommend piloting financial compensation for living kidney donors at a transplant centre in England.

## Introduction

There is a global shortage of organs for donation and each year between 250 and 400 people in England die whilst on the transplant wait list for a kidney. This is likely a significant underestimate given that this number does not include those removed from the wait list because they become too ill to receive a transplant. For example, between 1 April 2021–31 March 2022, 407 patients were removed from the kidney transplant wait list in the UK (NHS Blood and Transplant, 2022). In 2019, the Human Tissue Act 2004 (HT Act) was amended to allow England to adopt an opt-out system of organ donation, which was subsequently passed as The Organ Donation (Deemed Consent) Act 2019 and implemented in May 2020. This amendment aims to change the way donor consent is given for transplantable organs and tissues. Its intention is to increase the number of organs available for transplantation to save lives and improve the quality of life of those on the wait list. It was estimated by the United Kingdom (UK) Government that this amendment would save 700 lives per year (Dyer 2019). Despite these intentions, this amendment is unlikely to make a significant difference to the number of available organs.

Currently, there is no definitive evidence to suggest that merely adopting an opt-out system will increase the pool of available organs (Etheredge 2021). Nevertheless, even if the pool of organs were to increase, it is not necessarily a panacea. Spain, though not strictly an opt-out system because it does not have an opt-out register (Etheredge 2021), is considered the gold-standard system for organ transplantation. But despite their success, Spain still has an insufficient number of organs, a growing kidney transplant wait list, and patients still die waiting for a transplant (Crespo et al. 2021). Kidney transplant wait lists continue to increase despite improving infrastructure, education, and the adoption of opt-out systems. Because only around 1% of people who die each year in the UK are eligible to donate their organs (NHS Blood and Transplant, 2022), it is becoming increasingly necessary to consider alternative approaches to increase the number of available organs for transplant.

Other proposals to address the kidney shortage include xenotransplantation, which describes the cross-species transplantation of cells, tissue, or organs; despite significant recent progress it has not yet moved onto formal human clinical trials. However, it is not unreasonable to believe that kidney xenotransplantation using transgenic pigs could one day contribute to addressing the global shortage of kidneys. Whether it will demonstrate clinical efficacy remains undetermined until it has moved into the clinical trial phase (Cooper et al. 2021). There are also animal welfare concerns because of the need to breed and kill large numbers of transgenic pigs each year, and until formal clinical trials begin, the absolute risk of transmission of an infection—xenozoonosis—from a xenograft to the recipient remains unknown (Fishman 2018; Johnson 2022; Rodger and Cooper 2023).

Another proposed solution for the ongoing shortage is to introduce financial compensation for kidney donors to help address disincentives and in recognition of the goodness and generosity of the act^1^. The recent adoption of deemed consent indicates that there is an interest in making genuine improvements that will address the organ shortage. In addition, we have also seen the recent introduction of prioritising previous living kidney donors if they should require a transplant in the future (Zalewska 2018). These incremental changes in donation policies could arguably be interpreted as a shift in the donor-recipient relationship from being purely altruistic to a relationship that is more reciprocal. Because of this, we think that now is the right time to reopen—or reconsider—the debate and discussion about financial compensation. Here, we make the case for financially compensating living kidney donors in England who opt-in to receive it, whilst still permitting individuals to donate without receiving any compensation. Financial compensation should be understood as awarding a donor a predetermined set amount of money for their donation. Financial compensation should be distinguished from reimbursement where a donor is allowed to claim for any expenses that occurred during the living donor assessment process.

First, we explain why financially compensating living kidney donors in England should be considered ethically acceptable and identify one of the purported benefits—an increase in the number of available kidneys for transplantation. Second, we describe the potential economic benefits that include significant cost savings that can be redistributed into the prevention of kidney disease and reducing kidney disease disparities. We argue that financial compensation of around £35,000 for living kidney donors could be considered fair, effective at addressing common disincentives, and ethically permissible. We then address three common objections to our proposal. Though we focus on England, we do not see any reason why it could not be similarly effective anywhere else in the UK given their shared organ donation and transplantation infrastructure. Although we believe that financial compensation could, in principle, be effective in other high-income countries, given cultural nuances and the differences in healthcare systems and infrastructure we limit our positive case to England. One of our aims is to promote the idea of financial compensation beyond that of a mere hypothetical academic argument, therefore, we suggest that financial compensation should ideally be tested at one predetermined transplant centre in England—preferably one that routinely undertakes a higher volume of renal transplants annually. Following this approach would mean that the living donor team is more experienced in donor work-up and thus able to provide a more nuanced assessment of the donor’s motives. In addition, only relying on one pilot project would allow a better-controlled environment which will help to mitigate any foreseen or unforeseen risks to the donor.

## Background

On the 27th of July 1989, the Human Organ Transplants Act 1989 (HOTA) was passed, making it illegal to make or receive any payment for an organ intended for transplantation. This new legislation followed a kidney transplant scandal that occurred in England in 1989. The scandal involved three doctors—Michael Bewick, Michael Joyce, and Raymond Crockett—at a private London hospital who had been discovered to have transplanted four kidneys that had been purchased from abroad. In one case, a 33-year-old printing worker from Turkey sold his kidney for £2,500 following an advertisement in a Turkish newspaper. He was motivated to do so to help pay for medical treatment for his eight-year-old daughter (Frow 1997). Crockett, who was responsible for procuring the organs and arranging the payments had failed to ensure that the four men understood the risks and possible complications of the surgery, and establish valid informed consent (Tannenbaum 2014).

Although those involved in the scandal were rightly reprimanded, the speed with which the very notion of payment for organs was condemned and prohibited was surprising. More importantly, as Janet Radcliffe Richards (2003) points out, no consideration was given to the potential benefits that a regulated system might produce and balancing them against the potential for harm. The 1989 organ scandal clearly highlighted the potential for abuse, but it would be a mistake to presume that payment or compensation necessarily entails such things.

The HOTA was repealed and replaced by the HT Act following the Alder Hey Children’s Hospital organs scandal.^2^ The new legislative framework moved away from an ‘absence of objection’ system towards one that is solely focused on ensuring informed consent where human organs or tissue are concerned. Sections 32 and 33 of the HT Act continued to prohibit commercial dealings in human materials or the exchange of a reward where a living donation takes place. As a result, all living kidney donors must undergo an interview with the Human Tissue Authority (HTA). The purpose of this interview is to ensure that the donor has not received a reward and that the donor has consented to the process without any pressure from prospective recipients—most importantly the HTA interview aims to ensure that the donor has not been coerced (Human Tissue Act, 2004). In England, kidney donors, however, are eligible to claim financial reimbursement from NHS England that will cover their loss of earnings and other relevant expenses up to £5,000. The purpose of this reimbursement is to ensure that the impact on the living donor is cost-neutral and based on the premise that there should be no financial incentive to donate an organ (Department of Health, 2008)^2^.

## A monopsony system

We are not the first to propose a monopsony system, which describes a market in which there is one ‘buyer’—in this case the National Health Service (NHS)—who would continue to distribute kidneys on clinical need. One of the most well-known proposals for this was by Erin and Harris (2003). Here, we build on their case, providing additional evidence and argumentation in defence of it, specifically for living kidney donation in England. One of the primary differences with our proposal is that we do not think that financial compensation should be the default, rather, an option that a living kidney donor would have the freedom to opt-in to receive. Our preference for an opt-in system is rooted in the fact that the acceptance of financially compensating living donors is heavily clouded by strong moral feelings. Although we acknowledge that individuals should be allowed to let their moral values drive their decision-making, we cannot deny or ignore the fact that hundreds of people continue to die each year in England due to a lack of transplantable organs. We, therefore, argue that it would be more sensible to allow prospective donors the opportunity to actively choose whether they would like to receive compensation or not.

## Financially compensating kidney donors

We believe that it is ethically permissible for living kidney donors to be financially compensated for their associated costs. Currently, donors can be reimbursed for travel expenses and loss of earnings, but we suggest that other costs should be considered. A common example is the costs incurred when a loved one accompanies the donor on the day of donation. The costs will typically include wages lost, travel, and accommodation (if they do not live close to the transplant centre, which is often the case). Furthermore, these should include any health risks—however small—and psychological costs like anxiety, worry, and stress, thereby addressing some of the disincentives to donation. One obvious disincentive is any morbidity and mortality risks associated with nephrectomy. However, it remains a safe procedure with a perioperative mortality rate of between 0.02 − 0.04% (Garcia-Ochoa et al. 2019), and long-term mortality risk does not differ significantly (Park, 2021). But despite the low risk this still acts as a disincentive (McCormick et al. 2019). Other disincentives may include postoperative pain and discomfort, uncertainty about the long-term health impact of donating a kidney, and concerns about whether the kidney they donated might be needed by a family member in the future (McCormick et al. 2019). Financially compensating kidney donors is one feasible way to address these disincentives, and there is evidence that it can be considered acceptable without being perceived as an undue inducement (Gordon et al. 2015). In the United Halpern et al. (2010) found that amongst individuals medically eligible to donate the payment for kidney donation increased the probability of donating but it did so equally across all the different income levels; therefore the results suggest that financial payment does *not* especially act as an undue inducement for lower-income persons. Moreover, there is no plausible overriding ethical reason why a kidney donation should *only* be an altruistic or a supererogatory gift with no expectation of reward. It may be desirable for a living kidney donation to be altruistic but there is no plausible justification for it to be a *necessary* requirement (Moorlock et al. 2014). For example, it is acceptable to permit related-living donations that are not usually strictly altruistic, in that they are motivated by self-interest—the preservation of the life of a loved one. Such donations are still generous and good, and whilst the expectation of some kind of reward—whether financial or otherwise—could be considered less good, it is *not* bad to do so.

The underlying principle is generally considered uncontroversial—paying or compensating people to do good things and accepting some risk for doing so neither necessarily diminishes the good nor makes payment immoral. We suggest that compensating kidney donors is not and should not—in principle—be viewed as any more controversial than this. For instance, the care given by a doctor, nurse, or allied health professional is not diminished, necessarily less genuine, or ethically compromised because they are paid to provide it. Not only do healthcare professionals derive satisfaction from the care they provide but they similarly recognise that without payment they could not do the good they do, and yet we do not recognise that as coercive. Providing donors understand that what they are doing is good and they can provide valid informed consent—which is what the current system already ensures—then financial compensation cannot diminish it.

As Erin and Harris (2003) have pointed out, everyone else—other than the donor—in the transplant process is either already being paid, or benefitting substantially in the case of the recipient. We must reflect on whether it is fair for an individual to absorb the burden of risks involved by undergoing surgery that has no therapeutic benefit, without receiving any compensation. Some donors will donate just for the satisfaction of giving a loved one potentially several years of additional life; nevertheless, in such cases, it is not obvious that receiving compensation would diminish the generosity of the act. In fact, providing the option of financial compensation may be a fairer way of responding to the risks a living kidney donor accepts.

Despite believing that financially compensating donors is ethically permissible, we would suggest that donors would need to opt-in to receive the compensation to avoid making donors who for various—ethical or ideological—reasons would not want to receive any financial compensation. Our proposal also seems to be well aligned with the *Montgomery v Lanarkshire Health Board* [2015] judgment as it would permit prospective donors to make a decision about whether they would prefer to receive financial compensation or not. Therefore, recognising the importance of patients making autonomous choices about their healthcare. When considering the ongoing shift towards patient-centred healthcare and shared decision-making, the argument might even be made that it could be perceived as paternalistic to deny donors the opportunity to opt-in to receive compensation for their donation.

One problem where financial compensation could have a significant role to play in helping to address disincentives and raising awareness of living kidney donation among minority ethnic groups. Minority ethnic patients in the UK wait much longer than white patients for kidney transplants because of proportionally higher demand, lower deceased donor consent rates, fewer willing living donors, and a significant minority lacking knowledge of living donation (London Assembly, 2019; Pisavadia et al. 2018). A donor is much more likely to find a tissue and blood match if they are from the same ethnic background and so this disparity can only be addressed by increasing the number of willing donors. It is possible that financial compensation could be one means of redressing this imbalance, resulting in more equitable outcomes for these patients by encouraging more minority ethnic individuals to become living kidney donors. When kidney transplant recipients in the UK were asked why family members were not able to become living kidney donors, minority ethnic participants were more likely than White participants to state that financial concerns had prevented it (Wong et al. 2020). Therefore, it is plausible to believe that financial compensation has a role to play in increasing the number of minority ethnic living kidney donors.

This proposal may be considered provocative, and we are by no means the first to make this kind of case (Radcliffe-Richards et al. 1998; Gill et al. 2002; Friedman 2006; Pattinson 2008; Cherry 2017; Timmins and Sque 2019; Sterri 2021; Semrau and Matas 2022; Becker et al. 2022), but this merely reflects the increasing discontent with the *status quo*. The rationale is, in fact, very similar to that given for adopting deemed consent and the opt-out system in England—increasing the pool of available kidneys for transplantation and saving the lives of potential organ recipients. If this would be the case, then there is, on balance, a case for adopting this practice. Financial compensation for living kidney donors could, in principle, result in a significant reduction in wait times for kidney transplants—perhaps from three years to just one. In one region of Iran, the average wait time for a kidney transplant has decreased to just over one year (386.22 days) and most of the kidneys are purchased within the government-regulated market (Malekshahi et al. 2020). Moreover, even if this were not the case it could at the very least lead to a reduced burden on dialysis services and help to prevent the premature deaths of people who become too sick to receive a transplant.

It is important to acknowledge that many people experience a profound and intuitive moral disgust at the notion of donating an organ in exchange for any financial reward. For example, Kass (1992) describes feeling repulsed by the idea, and that it fundamentally undermines the dignity of the donor or ‘seller’, despite agreeing that it would likely increase the supply. Kass, however, only emphasises the potential normative implications for the donor or ‘sellers’ dignity, whilst understating the violation of the dignity of its potential recipients. Reese and Pies (2023) argue that when the dignity of the recipient is considered equally alongside the dignity of the donor there is no compelling notion of dignity that permits donating altruistically but rules out receiving financial payment or compensation for doing so^3^.

## Economic benefits

Despite currently being illegal in England, it is important to consider the economic benefits and drawbacks of a system where living kidney donors receive financial compensation (Morris et al. 2022). In 2009–2010^4^, the NHS in England spent around £1.45 billion (~ 1.3% of its budget) on treating chronic kidney disease, or £1 in every £77 spent (Kerr et al. 2012). It has been conservatively estimated that kidney transplantation results in a cost saving of between £15,000 - £25,800 per year post-surgery when compared to dialysis^5^, with most cost savings occurring in the second and subsequent years (Kerr et al. 2012). NHS Blood and Transplant (2009) have estimated that over a 10-year period, they save £24,100 per year that a patient has a functioning kidney transplant. So, not only would there be a significant cost-saving to the NHS, but the earlier patients have access to a kidney transplant the quicker they can benefit from its short- and long-term health benefits (Tonelli et al. 2011)

We suggest that a tax-free figure of £35,000 could potentially be an acceptable amount of financial compensation for a living kidney donor in England; this is slightly above the 2022 median full-time annual income in the UK of approximately £33,000 (Office for National Statistics, 2022). We acknowledge that it would be important for any suggested figure to be tested against public opinion to ensure that it is perceived as fair and not as an undue inducement. Our suggested figure of £35,000 is slightly lower than the median lowest amount of financial compensation that was perceived as an undue inducement for family/friends and substantially lower than for a stranger in the United States (Gordon et al. 2015). Furthermore, a cost-benefit analysis conducted in the US showed that compensation of approximately $77,000 [~£60,000] could save 47,000 patients per year from suffering unnecessarily on dialysis or dying prematurely due to being unable to access a timely kidney transplant (McCormick et al. 2022). Financial compensation could also be combined with other considerations, like certain tax benefits for living kidney donors, and prioritisation for counselling, psychological therapies, and support services. Arguably, the amount is less important than trying to establish that it is, in principle, acceptable to financially compensate living kidney donors.

Adding the indicative costs of the transplant (£17,000 according to NHS Blood and Transplant) to the £35,000 of financial compensation to the donor we get a total first-year cost of £52,000. However, once factoring in cost savings over a 10-year period we end up with a total cost saving of ~£186,000 per kidney transplant patient. Our proposal, therefore, has the potential to bring about significant cost savings that could be utilised to tackle chronic kidney disease, which by 2040 is estimated to increase from the 16th to the 5th leading cause of years lost (Jager et al. 2019; Foreman et al. 2018).

There are also hidden social and financial costs to kidney disease, because when the primary earner in a family is no longer able to work or must work less due to the demands of dialysis, further compounding the existing inequalities in England (Caskey and Dreyer 2018; Plumb et al. 2021). Individuals from lower socioeconomic groups have been found to be more likely to develop kidney disease; progress more quickly to a more serious stage of kidney disease; have poorer survival rates on dialysis; be more likely to experience kidney transplant rejection; and more likely to die earlier from chronic kidney disease (Caskey and Dreyer 2018).

One possible outcome to consider is what happens if all living kidney donors decide to opt-in to receive financial compensation since this would then incur significant additional costs. In the UK, there were 1039 living donor kidney transplants in 2019-20, 422 in 2020-21, and 890 in 2021-22 (NHS Blood and Transplant, 2022a; NHS Blood and Transplant, 2022b). If we assume a total of 1000 living donor kidney transplants plus an additional 250, to factor in the number of people who die each year waiting for a kidney transplant we get 1250. This would amount to an additional outlay of at least £48 million per year^6^. This is not an insignificant cost, but it will be offset by the clinical benefits and cost savings associated with earlier transplantation and the subsequently improved morbidity and mortality rates. Moreover, for every year that someone on the transplant wait list is unable to work due to ill health, there is an estimated lost contribution to the UK economy of £70,000 (Department of Health & Social Care, 2023).

## Iran’s kidney donation program

Since 1988 Iran has operated a state-level market in kidneys and remains the only country in the world to do so. It is based on the premise that the best outcome for someone with end-stage kidney disease is a successful transplant and that it would increase the supply of kidney donors. We should emphasise here that we are *not* advocating replicating Iran’s approach, instead, we merely want to recognise one of its core benefits—the significant reduction in wait time for a kidney transplant (Moeindarbari and Feizi 2022). This means that every individual, irrespective of socioeconomic status has access to kidney transplantation.

Ultimately, the goal of any organ donation system should be to *ethically* secure the number of organs required to ensure that everyone that requires a transplant can receive one. The Iranian project demonstrates that paying donors can increase supply, but our concerns are whether kidneys are always being *ethically* sourced. A study of 60 kidney donors in Iran showed that the vast majority (78%) of donors were satisfied with donating their kidneys, but 22% regretted doing so (Khatami et al. 2015). Regret may be related to the negative outcomes associated with the poor preoperative and follow-up care provided, for example, one donor had hypertension and two had poorly defined cardiac disease. Despite no evidence of coercion, more subtle pressures have been identified (Fry-Revere et al. 2020), and there remain serious concerns about the quality of the informed consent process. In one sample of kidney donors, 60% had not been informed about the risks and benefits of kidney donation (Khatami et al. 2015). There are also increasing concerns regarding the long-term follow-up care of Iranian living donors (Mahdavi-Mazdeh 2012). It is, however, highly unlikely that this would be the case in England as all living donors are provided with a yearly follow-up assessment with a renal physician.

## Objections to financially compensating kidney donors

There are three common objections raised against the implementation of paying or compensating donors that we will address. First, vulnerable kidney donors from lower socioeconomic groups could be exploited. Second, given the vulnerable state (e.g. poverty) of some potential donors, compensation would act as a form of coercion. Third, financial compensation will lead to a ‘crowding out’ effect, resulting in fewer donors. Although some of these arguments take a variety of forms, they are ubiquitous in the scholarly literature and we address each in turn.

## Financial compensation is exploitative

One does not have to look far to find claims that any system that resulted in a donor receiving some form of financial payment for a kidney would result in the exploitation of vulnerable individuals (Danovitch and Delmonico 2008; Hughes 2009; Adair and Wigmore 2011; Greasley 2014). Exploitation is frequently argued not to be just a possibility but an inevitable outcome and therefore should not be permitted because of this risk.

It is worth first describing what we mean by exploitation and then assessing whether the NHS financially compensating an individual would necessarily meet the conditions required to be described as exploitative. There are two main kinds of exploitation: economic and moral exploitation. These are: (1) paying less than the fair market value, and (2) wrongly taking advantage of someone.

The first kind of exploitation is primarily monetary; the concern is that a donor would be exploited if they were compensated less than fair ‘market value’ that was not reflective of the costs accepted by the donor and any cost savings accrued as a result. So, to avoid claims of exploitation only means ensuring that any financial compensation is deemed fair—by the donor and the public. Although we have proposed compensation of £35,000, it is possible that given the long-term cost savings it provides, a higher amount could be justifiable whilst avoiding undue inducement. However, all we want to note here is that it is possible to identify a fair ‘market value’ and that, were this provided, then what we have proposed should not be understood as exploitative. Several scholars have attempted to derive what a ‘fair market value’ might be but estimates vary depending on the country and kind of system being proposed (Becker and Elias 2007; Held et al. 2018). In a monopsony there would only be one ‘buyer’—the NHS—and so absent of competition it would be necessary to ensure that the financial compensation is both fair and widely perceived as such.

The second kind of exploitation is the more common of the two and is seen as an inherent component of financially compensating donors. It describes the buyer as taking advantage of a vulnerable donor who may one day come to regret their decision. However, merely taking advantage of someone does not equate to exploitation; McLachlan (2021) gives the example of a divorce lawyer who takes advantage of a marital breakdown to earn money. What matters is whether there is an aspect of wrongness to the advantage and whether that wrongness is primarily rooted in it being unjust.

McLachlan (2021) provides an illustration of this: ‘To threaten someone with a gun and thereby steal, say, his watch is exploitation. To offer to buy a watch from someone is not necessarily exploitative’. Merely being a legally permissible option for individuals can hardly be construed as the equivalent of threatening someone; even if someone did not like the fact that someone has offered payment for some good or service it does not necessarily follow that it is exploitative. The case for exploitation is even weaker because the ‘buyer’ in our system is not actively offering to purchase kidneys, it is merely one option available for individuals who, on balance, would be willing to accept compensation for providing an individual and societal good.

A monopsony helps to avoid many of the concerning ethical issues that critics have toward any kind of payment for a kidney (e.g. an illegal market and a power imbalance between the buyer and vendor). As is already the case in England, it would remain illegal for an individual to arrange and pay someone for their kidney, and to take advantage of someone’s vulnerable state; in our proposal, the ‘buyer’ or NHS already has an established system for reimbursing costs to donors. They could only be accused of exploitation *if* they never paid the agreed compensation that the donor opted into receiving.

Concerns about regret because of exploitation may also be misplaced since this always remains a risk whether someone is financially compensated or not. Imagine that an altruistic (or non-directed) donor gives a kidney to a teenager who grows up to commit some atrocity and they now regret doing so. Doing something for altruistic motives does not necessarily protect someone from regret. Our proposed system of financial compensation for kidney donors, therefore, does not meet the conditions to be considered exploitative in either sense.

## Financial compensation is coercive

A second common objection made against the use of payment or compensation for kidney donors is that it is coercive (Koplin 2018; Caplan and Rhodes 2022). The HTA (2020) describes coercion to entail that ‘the will of the person required to act has been overborne such that they can no longer make an independent decision’. Coercive practices or policies are those that usually involve some kind of threat. A recent example of a coercive policy would include the threat of redeployment or job loss made against health and social care workers in England who objected to the government’s mandatory COVID-19 vaccination requirement (Rodger and Blackshaw 2022). However, the option to receive financial compensation for certain costs (e.g. psychological) for donating a kidney does *not* involve a threat. Whilst we would agree that a coercive kidney donation would be wrong, merely being paid or receiving financial compensation for doing something is not sufficient to be coercive. We are sympathetic to this concern given the documented cases of coercion that did involve a threat (Naqvi et al. 2007). However, given the existing safeguards in the organ donation and transplantation systems in England, we believe that objecting to our proposal on this basis is unwarranted.

Importantly, the potential for a coercive kidney donation already exists in the absence of any financial compensation. A subtle example of coercion can be present when an individual who is related to the recipient is considering being a living donor. The pressure or threat in such instances may not be explicit, but the ‘threat’ here could be understood as the potential loss of a loved one (Caplan and Rhodes 2022). Because if they are a match, not donating will likely entail either the suffering associated with continued dialysis or their death. Any perceived threat may not be external but from them themselves, or ‘internal coercion’ (Siegler and Lanto, 1992). It is common for unrelated donors to be perceived with a higher degree of scepticism, regarding their motivations, but the presence of coercion may be as or more likely when a donor is related to the recipient.

Some may be tempted to claim that being coerced into donating would increase if doing so involved the kind of financial compensation we have proposed. We are sceptical of this claim because the existing safeguards and processes would continue to ensure that coerced donors are identified and not permitted to donate. Each potential donor would continue to be assessed by an independent assessor—who is trained to identify coercion—from the HTA as is standard practice in England. Arguably, a monopsony system in England would reduce the incidence of coerced donors because *if* it increases the number of available kidneys, then it would reduce the incentives and motivation to do so i.e. the desperation to find a kidney for oneself or a loved one. A recent example of the safeguards working successfully was the identification of a criminal conspiracy where a wealthy Nigerian politician and his wife attempted to pay a 21-year-old Nigerian man to donate a kidney to their daughter. He was allegedly made to pose as a family member and was coached to provide false answers to clinicians at the Royal Free Hospital in London (Weaver 2023).

## Financial compensation will ‘crowd out’

The crowding out effect describes when a financial incentive may ‘crowd out’ altruistic donors and lead to an insufficient number of individuals willing to donate. In his influential 1970 book, *The Gift Relationship*, Titmuss (1997) explored financial incentives and their impact on the donation of blood. Titmuss found in his study that financial incentives crowd out more donors than they ‘crowd in’, i.e. attract. Becker and Posner (2009) point out that Titmuss ignored the fact that in the United States—at the same time—paid blood donation was producing more blood per capita.

The rationale for ‘crowding out’ is that financial incentives may reduce altruistic motivation, thereby crowding out pro-social behaviour, and leading to a reduction of donors. A systematic review of the incentivisation of blood donation—of the albeit limited evidence available—showed that incentives, which were *not* always financial had no impact on the likelihood of donation, thus *prima facie* supporting Titmuss’ claim (Niza et al. 2013). Titmuss’ argument has been influential and there are concerns that the option of financial compensation could crowd out otherwise willing donors (Koplin 2018; Rothman and Rothman 2006).

However, we are sceptical of comparing the effects of incentives in a regular low-stakes blood donation with a high-stakes one-off kidney donation. As Semrau (2019) has pointed out, the stakes and the size of the incentive matter—the effects of crowding are only observed when the incentives are small, and its effect disappears when the incentive is increased. The financial compensation we advocate does not meet these criteria and therefore is highly unlikely to be subject to ‘crowding out’. We maintain that compensation should not be the default, thereby preserving the opportunity to be an ‘altruistic’ donor, and further reducing the risk of a crowding out effect. Becker et al. (2022) in their recent economic analysis, building on their previous work discovered that, paradoxically, payment makes the crowding out of altruistic donors less likely. Nevertheless, even if there is ‘altruistic’ donor crowding out, Iran provides evidence—not just theoretical conjecture—that a system where donors receive payment increases the total number of available kidneys.^53^ Furthermore, in a cross-sectional study of 343 participants who were medically eligible to donate, Halpern et al. (2010) found no evidence that the possibility of payment reduced the willingness of individuals to donate a kidney for altruistic reasons. An obvious limitation, however, is that there is often a disparity between what people say and what they actually do in the real world, and the only way to assess this is by piloting financial compensation. Ultimately, altruistic-based systems, despite their numerous benefits, are no longer fit for purpose and if we are to address the kidney shortage, we must consider alternative approaches.

## Conclusion

We have argued that the rationale given for the implementation of the opt-out system of organ donation in England equally applies to the introduction of financial compensation for living kidney donors. This is because it will make more kidneys available and potentially improve the quality of life for hundreds of people who die each year who need a kidney transplant. The adoption of opt-out—or other systems—has been shown to be unable to address the organ shortage and this shows no signs of changing. The monopsony approach we propose has several potential benefits that include saving hundreds of lives each year and improving the quality of life of those on the transplant wait list; a significant reduction in the kidney transplant wait list times; the prevention of avoidable deaths; cost savings that can be directed to proactively address the causes of kidney disease; the provision of fair financial compensation for costs to the donor (e.g. psychological); and helping to raise awareness of living donation. If financial compensation does result in a larger pool of available kidneys, it will also help to disincentivise attempts to coerce kidney donors. Furthermore, because financial compensation would not be the default, donors would be required to opt-in to receive it. Finally, we addressed three common objections and found them insufficient to rule out adopting financial compensation for living kidney donors.

## Footnotes

1 It should be noted that our proposal is solely aimed at living kidney donation, and we do not necessarily believe that the same arguments can be applied to living liver donation. Our justification for this approach is the following. Firstly, that the medical and psychosocial risk associated with liver donation is significantly higher than living kidney donation and therefore these two practices cannot be easily compared (Dew et al. 2017). Secondly, living kidney donation is grounded in data and evidence that has been captured over a long period and it has demonstrated that kidney donation is an acceptable practice with acceptable risks. Unfortunately, at this stage a similar data set is not available for living liver donation and as such it is rightly approached with greater caution in clinical practice.

2 The provision of financial reimbursement is an attempt to address disincentives to living organ donation, though as we go on to argue, this does not go far enough.

3 In a similar vein, Meilaender (2006) understands organ donation through a dichotomous lens: either a gift or a commodity. He is concerned that if ‘we learn to regard our bodies simply as collections of organs potentially useful to others, we are in danger of losing any close connection between the person and the body’. See Hippen (2006) for a detailed critique of Meilaender’s philosophical argument and assumptions.

4 The most detailed economic data exists for this period and so all costs discussed, unless otherwise indicated, are from this period.

5 The cost of dialysis has been estimated to be between £27,000 - £30,800 per year.

6 A crude estimate of £35,000,000 from 1000 (average annual living donors) x £35,000 (compensation only) and £13,000,000 from 250 (new donors) x £52,000.
